# The effect of melatonin and vitamin C treatment on the experimentally induced tympanosclerosis: study in rats^☆^

**DOI:** 10.1016/j.bjorl.2016.06.008

**Published:** 2016-07-20

**Authors:** Sema Koc, Halil Kıyıcı, Aysun Toker, Harun Soyalıç, Huseyin Aslan, Hakan Kesici, Zafer I. Karaca

**Affiliations:** a Antalya Education and Research Hospital, Department of ENT Head and Neck Surgery, Antalya, Turkey; b Mevlana University, School of Medicine, Department of Pathology, Konya, Turkey; c Necmettin Erbakan University, School of Medicine, Department of Biochemistry and Clinical Biochemistry, Konya, Turkey; d Gaziosmanpasa University, School of Medicine, Department of Otorhinolaryngology, Tokat, Turkey; e Gaziosmanpasa University, School of Medicine, Department of Histology and Embryology, Tokat, Turkey

**Keywords:** Tympanosclerosis, Melatonin, Vitamin C, Total antioxidant status, Timpanoesclerose, Melatonina, Vitamina C, Capacidade antioxidante total

## Abstract

**Introduction:**

The ethiopathogenesis of tympanosclerosis has not been completely under- stood yet. Recent studies have shown that free oxygen radicals are important in the formation of tympanosclerosis. Melatonin and Vitamin C are known to be a powerful antioxidant, interacts directly with Reactive Oxygen Species and controls free radical-mediated tissue damage.

**Objective:**

To demonstrate the possible preventative effects of melatonin and Vitamin C on tympanosclerosis in rats by using histopathology and determination of total antioxidant status total antioxidant status.

**Methods:**

Standard myringotomy and standard injury were performed in the middle ear of 24 rats. The animals were divided into three groups: Group 1 received melatonin, Group 2 received vitamin C, and Group 3 received saline solution.

**Results:**

The mean values of total antioxidant status were similar in the all study groups before the treatment period. The mean values of total antioxidant status were significantly higher in the melatonin and vitamin C groups compared to control group but vitamin C with melatonin groups were similar after the treatment period (*p* < 0.001). Minimum and maximum wall thicknesses were lower in the melatonin and vitamin C groups compared to the control group but the differences were insignificant.

**Conclusion:**

Melatonin increases total antioxidant status level and might have some effect on tympanosclerosis that develops after myringotomy.

## Resumo

**Introdução:**

A etiopatogênese da timpanoesclerose (TE) não foi ainda totalmente esclarecida. Estudos recentes têm demonstrado que os radicais livres de oxigênio são importantes na formação de TE. Melatonina e vitamina C são conhecidas por serem poderosos antioxidantes, interagirem diretamente com espécies reativas de oxigênio (ROS) e controlar danos em tecidos mediados por radicais livres.

**Objetivo:**

Demonstrar os possíveis efeitos preventivos da melatonina e da vitamina C na TE em ratos usando histopatologia e determinação da capacidade antioxidante total (CAT).

**Método:**

Miringotomias padronizadas foram realizadas na orelha média de 24 ratos. Os animais foram divididos em três grupos: o Grupo 1 recebeu melatonina, o Grupo 2 recebeu vitamina C, e o grupo 3 recebeu solução salina.

**Resultados:**

Os valores médios de CAT foram semelhantes em todos os grupos de estudo antes do período de tratamento. Os valores médios de CAT foram significativamente maiores nos grupos que receberam melatonina e vitamina C em comparação com o grupo de controle, mas os grupos vitamina C e melatonina foram semelhantes após o período de tratamento (*p* < 0,001). As espessuras mínimas e máximas de parede foram menores nos grupos melatonina e vitamina C, em comparação com o grupo controle, mas as diferenças não foram significativas.

**Conclusão:**

A melatonina aumenta os níveis de CAT e pode ter algum efeito sobre a TE que se desenvolve após a miringotomia.

## Introduction

Tympanosclerosis (TS) is a condition that may affect both the tympanic membrane and the middle ear mucosa. It is characterized histologically by an increase in collagenous fibers, decreased vascularization and cell formation hyalinization calcification and degeneration of the collagen layer.^1^, ^2^ TS is usually identified as white chalky patches either in the middle ear mucosa or in the tympanic membrane (TM). At the beginning of the pathology it consists of cheese-like masses of tympanosclerotic material and by the time similar to bone it gets harder.^3^ The ethiopathogenesis of TS has not been completely understood yet. Recent studies have shown that free oxygen radicals are important in the formation of TS in traumatized TM and antioxidant agents decrease or prevent myringosclerosis and TS after experimental myringotomy.^4^, ^5^, ^6^, ^7^

Melatonin (N-acetyl-methoxytryptamine), an indolamine derived from tryptophan, is mainly produced in the mammalian pineal gland but it is also produced in the other organs.^8^ It has been shown to act as a potent anti-inflammatory, antioxidant and free radical scavenger, protecting against a number of radical species. Various studies have established that due to these features melatonin decreases sclerosis.^9^, ^10^, ^11^ Vitamin C is known to be a powerful antioxidant, interacts directly with Reactive Oxygen Species (ROS) and controls free radical-mediated tissue damage.^12^

The aim of this study was to investigate and compare the possible preventative effects of melatonin and vitamin C on TS in rats, which had TM myringotomy and a standard injury in the middle ear, by using histopathology and determination of total antioxidant status (TAS).

## Methods

International Standards for the care of laboratory animals were followed and the protocol of the study was approved by the responsible local ethical committee.

### Animal maintenance

This study was carried out in compliance with the guidelines for animal experimentation at the University of Gaziosmanpasa. All animal care and procedures were performed humanely. Twenty-four Wistar-Albino type rats, weighing 250–300 g, were observed for 15 days in the animal care laboratory. Any animal that showed signs of external or middle ear infection and possibility of underlying diseases were excluded from this study. Animals were housed in a room maintained at a temperature of 23° ± 3 °C with 12 h light and 12 h darkness and with 13–18 air changes per hour.

### Operating procedures and experimental design

All interventions were performed under sterile conditions. After anesthesia with intramuscular ketamine hydrochloride 30 mg/kg, under the otomicroscope, through an aural speculum, the myringotomy was performed similarly on both ears of rats, with a radial orientation in the upper posterior quadrant of the TMs, length approximately 3–4 mm. Subsequently, a standard injury was formed in the middle ear. They were divided into three groups (Groups 1, 2, and 3). Eight rats (Group 1) received 5 mg/kg melatonin via orogastric gavage, eight rats (Group 2) received 75 mg/kg vitamin C via orogastric gavage, eight rats (Group 3) received saline (0.9% NaCl) solution via orogastric gavage; this was continued for 10 days. The amount of the melatonin and vitamin C supplement were determined with reference to the literature.^9^, ^13^

### Biochemical analysis

For biochemical analyses blood samples were collected at the first day from the tail veins of the rats when they were anesthetized prior to myringotomy and at the 28th day from intracardiac route again when they were anesthetized prior to scarification.

### Total antioxidant status (TAS)

Total antioxidant status levels were measured using commercially available kits (Rel Assay). The novel automated method is based on the bleaching of characteristic color of a more stable ABTS (2.2′-azino-bis[3-ethylbenzothiazoline-6-sulfonic acid]) radical cation by antioxidants. The commercial kit work colorimetric method is based on 660 nm absorbance. The results were expressed as mmol Trolox equivalent/L.

### Tissue collection and histopathological analysis

On the 28th day, all animals were euthanized painlessly following administration of high dose (80 mg/kg) intraperitoneal pentobarbital. The temporal bones of the animals were removed, enumerated, and the middle ear cavity, tympanic membranes, and external ear canals were isolated. The specimens were fixated within 4% formaldehyde, and then decalcified using 0.1 moL/L Ethlenediamine Tetra-acetic Acid (EDTA). Following tissue processing and blocking, specimens were cut to obtain 3 μm thick samples. Hematoxylin–Eosin (H&E) and Masson's trichrome stains were used for histopathologic evaluation. Thickness of lamina propria at the middle ear was measured by a graded ocular, under Olympus BX53 light microscope. Exudates were not included in thickness of lamina propria. When available, thickness of tympanic membrane was measured by the same technique.

### Statistical analysis

All statistical analyses were performed using SPSS for Windows version 15 (SPSS, Chicago, IL, USA). The Kolmogorov–Smirnov test was used to evaluate whether the distribution of variables was normal. The Student *t* or Mann–Whitney *U* tests were used to compare continuous variables between the two groups. Continuous variables were presented as mean (standard deviation [SD]) or as median (interquartile range [IQR]). A *p*-value of less than 0.05 was considered to be statistically significant.

## Results

**Figure 1:**
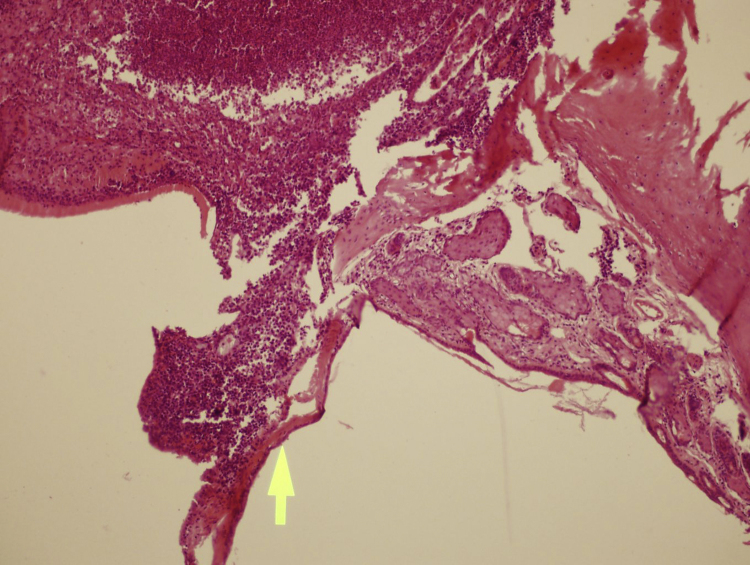
There is no significant tympanosclerosis (arrow) in this sample of control group; with moderate otitis media (left side of arrow). H&E stain, 100× magnification.

**Figure 2:**
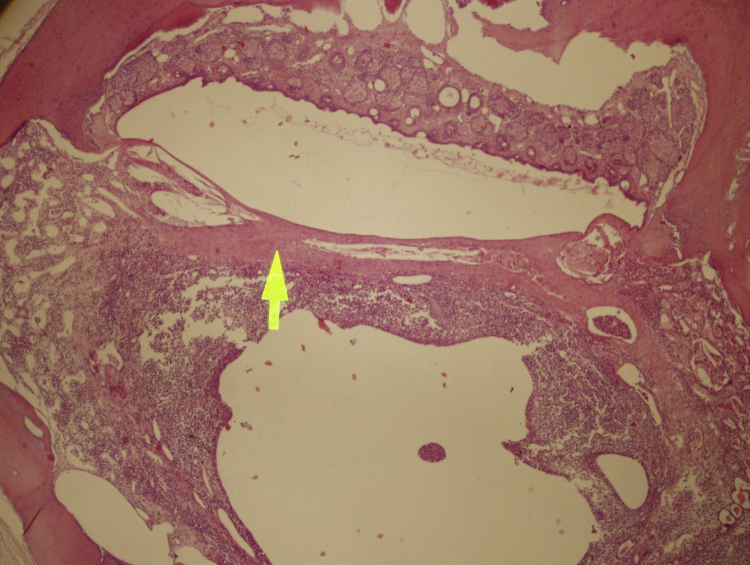
Mild tympanosclerosis (arrow) and mild otitis media (below the arrow) are demonstrated on a sample of melatonin group. H&E stain, 100× magnification.

The mean values of TAS were similar in the all study groups before the treatment period. The mean values of TAS were significantly higher in the melatonin and vitamin C groups compared to control group but vitamin C with melatonin groups were similar after the treatment period (*p* < 0.001) (Table 1). Minimum and maximum wall thicknesses were lower in the melatonin and vitamin C groups compared to control group but the differences were insignificant. Minimum and maximum wall thicknesses were similar in the melatonin and vitamin C groups (Table 1) (Figure 1, Figure 2).

**Table 1:** Total antioxidant status and wall thickness in the study groups.

	Control (*n* = 8)	Vitamin C (*n* = 8)	Melatonin (*n* = 8)	*p*
TAS before the treatment (mmoL Trolox Eq/L)^a^	2.97 ± 0.86	2.51 ± 0.39	3.20 ± 0.91	0.199
TAS after the treatment (mmoL Trolox Eq/L)^a^	2.51 ± 0.64	5.68 ± 0.28	5.46 ± 0.23	<0.001^c^
Minimum wall thickness^b^	2.35 (1.25–4.5)	2.0 (1.0–6.9)	2.0 (1.25–3.6)	0.946
Maximum wall thickness^a^	38 ± 27	33 ± 26	32 ± 17	0.847

TAS, total antioxidant status.

a Values are presented as mean ± standard deviation.

b Values are presented as median and interquartile range (Q1–Q3).

c There were statistically significant difference between control with melatonin groups (*p* < 0.001) and control with vitamin C groups (*p* < 0.001) but vitamin C with melatonin groups was similar.

## Discussion

Our study revealed that systemic administration of melatonin reduced or inhibited the formation of TS by acting as a free radical scavenger in rats which had myringotomy and formation of a standard injury in the middle ear. As far as we know, this is the first study in the literature to evaluate the effectiveness of melatonin in prevention of TS.

Reactive Oxygen Species are oxygen-containing molecules that are produced during normal metabolism under aerobic conditions. ROS include superoxide anion radical (O_2_•^−^), hydrogen peroxide (H_2_O_2_), hydroxyl radical (•OH) and single oxygen (^1^O_2_).^14^ These oxygen radicals are considerably reactive and posses the capacity of causing irreversible cellular destruction. The antioxidant systems contain enzymatic and non-enzymatic mechanisms against the harmful effects of the endogenous ROS products.^15^ Enzymatic system involves Superoxide Dismutase (SOD), Catalase (CAT) and Glutathione Peroxidase (GSH-Px). Ascorbic acid, glutathione, *β*-carotene, tocopherols, and uric acid can be included as non-enzymatic defense systems.^16^ Formation and elimination of free oxygen radicals are in a balance known as oxidative balance. As long as the oxidative balance is maintained, free oxygen radicals do not harm the organism. When the oxidative balance is corrupted, free oxygen radicals elevate and tissue damage occurs, this final situation is called as oxidative stress.^17^ It is possible to measure one by one the antioxidant parameters in serum. Antioxidant parameters have additive effects, thus individual values may not completely exhibit the total antioxidant status. Therefore TAS measurement is more appropriate procedure to assess antioxidative status.^18^

Tympanosclerosis is caused by recurrent acute otitis media, treatment of serous otitis media with ventilation tube insertion into the TM, chronic otitis media, immunological hypersensitivity reaction, genetic tendency, or trauma. Recent studies have investigated formation of myringosclerosis after myringotomy and pointed out a relationship between the development of TS and ROS.^1^, ^4^, ^5^ When compared to 10% in ambient air, approximately 5% oxygen concentration in the middle ear cavity is much lower than that in ambient air. Myringotomy leads to an increase of oxygen concentration in the middle ear cavity, resulting in a relatively hyperoxic condition.^19^ Hyperoxia causes formation of ROS thereby might provoke inflammatory process. The increase of ROS and the impairment of antioxidant defense mechanisms cause tissue damage involving fibrosis, hyalin degeneration, accumulation and aggregation of calcium and phosphorus forming sclerotic deposits. ROS scavengers have been shown to decrease the inflammatory reaction which also decrease the adhesion formation.^4^, ^5^, ^6^, ^7^ Therefore, antioxidant treatment may be hypothesized to reduce TS formation and reformation.

Melatonin is a potent antioxidant and free radicals scavenger.^9^ Both direct and indirect antioxidant effects of melatonin have been reported. Melatonin has an indirect antioxidant effect by inducing SOD and GSH-Px activities. By scavenging oxygen-derived free radicals, such as hydroxyl and peroxyl radicals, melatonin discloses its direct antioxidant effect.^14^ In addition, melatonin prevents from sclerosis by inhibiting aggregation and secretion of platelets, prostaglandin synthesis, and fibroblast proliferation.^20^ Rosa et al.^21^ have reported that melatonin reduces destruction and fibrosis caused by oxidative stress by means of elevating antioxidant enzymes such as SOD and GSH-Px in cirrhotic rats. Koc et al.^11^ have examined effects of melatonin on oxidative stress and adhesion in rats after laparotomy and standard injury on right uterine horn. In the melatonin given group they have found adhesion to be less, SOD and CAT activity to be high and Malondialdehite (MDA) level to be low. In another study melatonin has shown to prevent peritoneal adhesion by reducing oxidative stress.^9^

A curative treatment for myringosclerosis (MS) and TS has not been identified yet. Various antioxidants have been used in treatment and proved to reduce MS.^21^ Emir et al.^22^ have stated that ginkgo biloba extracts, which have antioxidant and anti-inflammatory effects, reduced or inhibited MS in rats who had myringotomy. Dundar et al.^12^ used ascorbic acid in myringotomized rat ears and showed that ascorbic acid reduced the occurrence of MS both in otomicroscopic and histopathologic examinations. Karlidag et al.^23^ have evaluated oxidative stress and TS in 65 patients who underwent tympanoplasty or tympanoplasty together with mastoidectomy. In the patients with TS levels were found to be high and CAT activity low as compared to the patients without TS, whereas, no difference was found between groups in SOD activity. Kazikdas et al.^5^ have demonstrated that alpha tocopherol reduces oxidative stress and MS by means of biochemical analysis, otomicroscopic, tympanometric and histopathologic assessments.

In the current study, effect of melatonin and vitamin C on TS was assessed in myringotomised and middle ear mucosa damaged rats by using histopathology and determination of oxidative status via measurement of TAS levels. In this trial, it was shown that the mean values of TAS were similar in the all study groups before the treatment period. The mean values of TAS were significantly higher in the melatonin and vitamin C groups compared to control group but vitamin C with melatonin groups were similar after the treatment period. Minimum and maximum wall thicknesses were lower in the melatonin and vitamin C groups compared to the control group but the differences were insignificant. Minimum and maximum wall thicknesses were similar in the melatonin and vitamin C groups.

## Conclusion

This study suggests that the administration of melatonin increases TAS level and might have some effect on TS that develops following myringotomy, but these observations are not statistically significant. Nevertheless, further experimental studies on large number of subjects using melatonin and/or vitamin C in high doses and for various periods should be designed to affirm the effects of melatonin and/or vitamin C on TS induced by myringotomy.

## Conflicts of interest

The authors declare no conflicts of interest.
